# Unravelling the Antimicrobial, Antibiofilm, Suppressing *Fibronectin Binding Protein A* (*fnba*) and *cna* Virulence Genes, Anti-Inflammatory and Antioxidant Potential of Biosynthesized *Solanum lycopersicum* Silver Nanoparticles

**DOI:** 10.3390/medicina60030515

**Published:** 2024-03-21

**Authors:** Alsayed E. Mekky, Ahmed E. M. Abdelaziz, Fady Sayed Youssef, Shymaa A. Elaskary, Aly A. Shoun, Eman A. Alwaleed, Mahmoud Ali Gaber, Abdulaziz A. Al-Askar, Alhadary M. Alsamman, Abdullah Yousef, Gehad AbdElgayed, Reda A. Suef, Mohamed A Selim, Ebrahim Saied, Mohamed Khedr

**Affiliations:** 1 Botany and Microbiology Department, Faculty of Science, Al-Azhar University, Nasr City, Cairo 11884, Egyptalsamman557@gmail.com (A.M.A.); redasuef@azhar.edu.eg (R.A.S.); mohamedselim@azhar.edu.eg (M.A.S.); hema_almassry2000@azhar.edu.eg (E.S.); mohamedkhedr.221@azhar.edu.eg (M.K.); 2 Botany and Microbiology Department, Faculty of Science, Port-Said University, 23 December Street, P.O. Box 42522, Port-Said 42522, Egypt; ahmed.abdelaziz@sci.psu.edu.eg; 3 Pharmacology Department, Faculty of Veterinary Medicine, Cairo University, Giza 12211, Egypt; fadyalsalhany@cu.edu.eg; 4 Medical Microbiology and Immunology Department, Faculty of Medicine, Menoufia University, Shibin El-Kom 32511, Egypt; 5 Microbiology and Immunology Department, Faculty of Pharmacy, El Salehey El Gadida University, El Saleheya El Gadida 44813, Egypt; aly.shoun@sgu.edu.eg; 6 Botany and Microbiology Department, Faculty of Science, South Valley University, Qena 83523, Egypt; eme_biologist@sci.svu.edu.eg; 7 Botany and Microbiology Department, Faculty of Science, King Saud University, P.O. Box 2455, Riyadh 11451, Saudi Arabia; aalaskara@ksu.edu.sa; 8 Basic & Medical Sciences Department, Faculty of Dentistry, Alryada University for Science & Technology, Sadat 32897, Egypt; abdullah.yousef@rst.edu.eg; 9 Integrated Molecular Plant Physiology Research, Department of Biology, University of Antwerp, 2020 Antwerp, Belgium; gehad.hegazygadabdelgayed@uantwerpen.be

**Keywords:** antibiotic susceptibility, anti-inflammatory, antioxidant, pathogenicity, phytochemical analysis, urinary tract infections

## Abstract

*Background and Objectives*: Urinary tract infections [UTIs] are considered the third most known risk of infection in human health around the world. There is increasing appreciation for the pathogenicity of Gram-positive and Gram-negative strains in UTIs, aside from fungal infection, as they have numerous virulence factors. *Materials and Methods*: In this study, fifty urine samples were collected from patients suffering from UTI. Among the isolates of UTI microbes, six isolates were described as MDR isolates after an antibiotic susceptibility test carried out using ten different antibiotics. An alternative treatment for microbial elimination involved the use of biosynthesized silver nanoparticles (AgNPs) derived from *Solanum lycopersicum* [*S. cumin*]. *Results*: The sizes and shapes of AgNPs were characterized through TEM imaging, which showed spherical particles in a size range of 35–80 nm, of which the average size was 53 nm. Additionally, the silver nanoparticles (AgNPs) demonstrated inhibitory activity against *Staphylococcus aureus* (OR648079), exhibiting a 31 mm zone of inhibition at a minimum inhibitory concentration (MIC) of 4 mg/mL and a minimum bactericidal concentration (MBC) of 8 mg/mL. This was followed by *Aspergillus niger* (OR648075), which showed a 30 mm inhibition zone at an MIC of 16 mg/mL and a minimum fungicidal concentration (MFC) of 32 mg/mL. Then, *Enterococcus faecalis* (OR648078), *Klebsiella pneumoniae* (OR648081), and *Acinetobacter baumannii* (OR648080) each displayed a 29 mm zone of inhibition at an MIC of 8 mg/mL and an MBC of 16 mg/mL. The least inhibition was observed against *Candida auris* (OR648076), with a 25 mm inhibition zone at an MIC of 16 mg/mL and an MFC of 32 mg/mL. Furthermore, AgNPs at different concentrations removed DPPH and H_2_O_2_ at an IC50 value of 13.54 μg/mL. Also, AgNPs at 3 mg/mL showed remarkable DNA fragmentation in all bacterial strains except *Enterococcus faecalis*. The phytochemical analysis showed the presence of different active organic components in the plant extract, which concluded that rutin was 88.3 mg/g, garlic acid was 70.4 mg/g, and tannic acid was 23.7 mg/g. Finally, AgNPs concentrations in the range of 3–6 mg/mL showed decreased expression of two of the fundamental genes necessary for biofilm formation within *Staphylococcus aureus*, *fnbA* (6 folds), and *Cna* (12.5 folds) when compared with the *RecA* gene, which decreased by one-fold when compared with the control sample. These two genes were submitted with NCBI accession numbers [OR682119] and [OR682118], respectively. *Conclusions*: The findings from this study indicate that biosynthesized AgNPs from *Solanum lycopersicum* exhibit promising antimicrobial and antioxidant properties against UTI pathogens, including strains resistant to multiple antibiotics. This suggests their potential as an effective alternative treatment for UTIs. Further research is warranted to fully understand the mechanisms of action and to explore the therapeutic applications of these nanoparticles in combating UTIs.

## 1. Introduction

Urinary tract infection (UTI), or bacteriuria, is the medical term for the presence of bacteria in the urine. A clinical infection may begin with 10^5^ bacteria/mL, but for epidemiological purposes, “notable” bacteriuria should be at least 10^5^ bacteria/mL in freshly voided urine [1]. Urinary tract infections are the most globally common infectious disease, affecting over 10% of the world’s 150 million people daily. Uropathogenic *Escherichia coli* (UPEC) is consistently identified as the primary causative agent in both complicated and uncomplicated urinary tract infections [2]. Other specific microbes implicated in the pathogenesis of complicated UTIs include *Enterococcus* spp., *Klebsiella pneumoniae*, *Staphylococcus aureus*, *Pseudomonas aeruginosa*, and *Candida* spp. Similarly, the development of uncomplicated urinary tract infections is significantly impacted by pathogens, including *Klebsiella pneumoniae*, Staphylococcus saprophyticus, *Enterococcus faecalis*, Group B Streptococcus (GBS), *Proteus mirabilis*, *Pseudomonas aeruginosa*, *Staphylococcus aureus*, and *Candida* species. The fundamental mechanism underlying UTI pathogenesis involves the adherence of specific virulence factors present in the bacteria in the urethra. This process is followed by the colonization and subsequent ascension of the pathogen into the bladder through its appendages, such as flagella and pili. Once the pathogens attach to the bladder, complex interactions between the host and the pathogen occur, progressing the disease to more advanced stages [3]. The cluster of virulence factors is an important and remarkable cause of multidrug resistance in the urinary tract [4]. MDR bacteria in urine complicates empiric treatment of urinary tract infections and increases the prevalence of infection [5]. Globally, the extraordinary emergence and proliferation of multidrug-resistant bacterial infections pose a hazardous public health problem and are occurring in growing communities [6]. Recently, the emergence of the MDR uropathogen has led to an increase in nosocomial urinary tract infections worldwide [7]. Many microbial species and isolates have not yet been identified or described [8]. Due to the continuous increase in microbial multidrug resistance and the limitations of antibiotic therapy, there is an urgent need to develop effective antimicrobial agents with new mechanisms of action [9]. Researchers interested in generating nanoparticles are currently focusing on developing new strategies and materials to produce green nanomaterials [10,11]. Nanotechnology is a developing field of science focused on the fabrication of materials with nanosizes. The synthesis and application of nanomaterials have minimal impact on nature and provide maximum social benefits, as highlighted in this state of thinking [12]. Noble metal nanoparticles, including silver, gold, and titanium, have garnered significant interest for their potential use in biological applications because of their diverse therapeutic qualities. Silver nanoparticles (AgNPs), known for their low toxicity to mammalian cells and broad-spectrum antibacterial activity, are among the most extensively researched nanoscale particles [13]. More research reflects that nanosized silver particles exert a strong antibacterial effect due to their high specific surface area [14]. Therefore, subsequent research has focused on nanoparticles as an alternative to conventional antimicrobial agents to control infections caused by multidrug-resistant bacteria (MDRB). Metal oxide nanoparticles have been shown to successfully function as promising antimicrobial agents against MDR isolates [15]. Essentially, many techniques, like mechanical, physical, and chemical, have been exploited to synthesize AgNPs. The biosynthesis approach, which makes use of microorganisms and plant extracts, has emerged as an environmentally benign synthetic method to overcome the drawbacks of physical and chemical methods [10,16]. Plant extracts provide an easy and advantageous way to create SNPs during biosynthesis since they are practical, easy to employ, rapid, affordable, and nontoxic. It is strongly advised to use plant extracts in order to produce AgNPs [17]. The outstanding antibacterial capabilities of plant materials can be attributed to their high content of secondary metabolites, such as enzymes, polysaccharides, alkaloids, tannins, phenols, terpenoids, and vitamins [18]. It is believed that the terpenoids and flavonoids found in the leaf extract aid in stabilizing the AgNPs [19]. Recently, there has been significant research into the biosynthesis of silver nanoparticles (AgNPs) using biological materials, such as plants and bacterial extracts as reducing agents, focusing particularly on their antibacterial activity. Nanomaterials are traditionally synthesized using one of two methods: i. “top-down” or ii. “bottom-up”. Using size reduction of bulk materials techniques, such as pulse wire discharge method, evaporation–condensation, ball milling, etc., the top–down approach produces nanoparticles. The bottom–up strategy involves the synthesis of nanoparticles (NPs) using chemical and biological mechanisms, wherein atoms self-assemble to form new nuclei that develop within tiny particles [20].

The common term “tomato”, *Solanum lycopersicum*, is chosen for this investigation. Its characteristics have led to the selection of this plant source. Tomatoes are a great source of several beneficial chemicals and antioxidants. They are staples in our daily diet. At the industrial level, tomatoes are used in large quantities to make purees and juices. *Solanum lycopersicum* has been shown to have antioxidant and anticancer properties. Certain phenolic chemicals are related to the function of an antioxidant. By lowering the body’s levels of free radicals, these substances guard against cell damage. The tomato plant was chosen for this study due to its nutritional characteristics and benefits [21].

AgNPs were produced by the oxidation of Ag^+^ to Ag^0^ by various biomolecules like flavonoids, carboxylic acids, phenolic acids, and aldehydes, along with proteins from plant extracts [22,23]. AgNPs are created through a simple, one-step process that does not produce toxic or expensive chemicals, making them safe, cheap, and environmentally friendly [24]. Recently, research has been intense on the biosynthesis of AgNPs with different shapes, molecular sizes, stability levels, and antibacterial efficacy in both plants and microorganisms [25]. In addition to being an effective material in reducing biofilm growth and preventing the spread of infectious diseases like nosocomial infections, urinary tract infections in children and adults are among the most common hospital-associated infections and are regarded as the third-most-known risk of infection regarding human health worldwide. AgNPs have been used laterally in medical clinics to reduce bacterial diseases [26]. Silver nanoparticles (AgNPs) have garnered significant attention, particularly in the field of biomedicine, due to their broad-spectrum and highly efficient antimicrobial and anticancer properties. Additionally, AgNPs have been explored for other biological activities, such as promoting bone healing and wound repair, enhancing the immunogenicity of vaccines, and exerting antidiabetic effects [27]. Understanding the biological mechanisms and potential cytotoxicity of AgNPs is crucial for optimizing their medical applications. The potential toxicity of AgNPs includes possible damage to various systems and organs in vivo, such as the skin, eyes, respiratory system, hepatobiliary system, central nervous system, urinary system, immune system, and reproductive system. One notable advantage of the proposed AgNPs over traditional silver-based antimicrobial agents is their enhanced efficacy at lower concentrations. The nanoscale size of AgNPs provides a larger surface area for interaction with microbial cells, thereby increasing their antimicrobial activity. Consequently, smaller amounts of AgNPs are needed to achieve the same antimicrobial effect as larger quantities of bulk silver compounds. This lower concentration requirement can reduce the potential toxicity to human cells, making AgNPs a safer option for clinical applications. Furthermore, the increased efficacy at lower concentrations can result in cost savings in both the manufacturing and application of AgNP-based products [28,29,30].

Recently, bacterial biofilms have been recognized as a public health concern. They are crucial to the survival of dangerous bacteria, which makes them increasingly resistant to medications [31]. So, AgNPs were also in competition with biofilm formation [32]. Our novelty in this research is to study the antimicrobial, antioxidant, and anti-inflammatory activity of tomato-leaf extract in comparison with green biosynthesized AgNPs and their effect on the expression of two fundamental genes of biofilm formation in *Staphylococcus aureus* at concentrations below its MIC.

## 2. Materials and Methods

### 2.1. Collection of Plant Material and Chemicals

*Solanum lycopersicum* leaves were collected from El-Qalubyia Government, Cairo, Egypt, (GPS, N: 30°11′39.34′′, E: 31°26′1.23′′). The chemicals and reagents, silver nitrate (AgNO_3_), dimethyl sulfoxide (DMSO), 2,2-diphenyl-1-picrylhydrazyl (DPPH), Tryptic Soy Broth (TSB), nutrient broth, agar, potato dextrose agar [PDA], purple p-iodo-nitrotetrazolium (INT, 0.2 mg/mL), NaOH, aluminum chloride, H_2_SO_4_, HCL, alcoholic KOH, MgCl_2_, methanol, ascorbic acid (Vitamin C), sodium phosphate buffer, and NaCl were of AR grade and procured from Sigma-Aldrich, Cairo, Egypt). All biological syntheses in the current study were achieved using distilled water (dis. H_2_O).

### 2.2. Preparation of Plant Extract and Biosynthesis of AgNPs

The tomato leaves were hand-pulled after being baked until completely dry. To prepare the extract, 50 g of powdered dry leaves were suspended in a solution of 50 mL of MeOH and 50 mL of H_2_O. The combination was then sonicated, and the mixture was filtered using Whatman filter paper no. 1. The residual water was removed through freeze-drying and stored at 4 °C, while the methanol was subsequently evaporated using a rotary [33]. The protocol of Oves et al. [34] was followed with some modifications. One mM of a 50 mL aq AgNO_3_ solution is frequently added to 5 mL of tomato-leaf extract in a 250 mL conical flask with vigorous agitation at 90 °C for 60 min in order to synthesize biogenic silver nanoparticles. A change in the mixture’s color served as our observation for the formation of AgNPs. The color of the silver nitrate solution changed to brownish yellow within 5 min, indicating that the bioreduction of the silver ions was rapid, confirming the preparation of AgNPs. Biosynthesized AgNPs were purified by centrifugation at 10,000 rpm for 10 min and were washed three times with DW to remove loosely attached biomolecules. Then AgNPs were transferred into a clean bottle in a dark place for further studies.

### 2.3. AgNPs Characterization

Different techniques were used to characterize biosynthesized silver nanoparticles. The change in color of the reaction mixture, following the reduction of Ag^+^ to Ag^0^ nanoparticles (NPs) by an extract derived from *S. cumin* leaves, was confirmed or assessed through a visual examination. UV light’s optical absorption characteristics were ascertained using a JASCOVIS-730 dual-beam spectrometer. The absorption spectrum was collected between 200 and 700 nm, with an additional wavelength increase of around 0.2 nm. The transmission electron microscope (EOL MODEL 1200EX, Ltd., Tokyo, Japan), running at 120 kV, was used to evaluate the micrographs of the samples. For this purpose, a drop of NPs solution was added to a carbon-coated copper grid and left to dry before being placed on a specimen holder. X-ray diffraction (XRD) characterization was conducted using a powder diffractometer (Ultima IV, Rigaku, Japan) equipped with a copper target, generating Kα1 radiation at a wavelength of 1.54060 Å. The 2θ range, which spans from 10 to 80 degrees, was measured. The 2θ/θ continuous mode was used for X-ray scanning, with a step size of 0.02 and a scanning rate of 2 degrees per minute. Both the voltage and the current in the tubes stay constant at 40 kilovolts (kV) and 40 milliamperes (mA). The average Ag-NP sizes were measured using the Debye–Scherrer equation [35] as follows:

D = Kλ/βcos θ

Here, D is the average particle size, K is the Scherrer constant (0.9), λ is the wavelength of X-ray radiation (0.154 nm), and β and θ are the half of maximum intensity and Bragg’s angle, respectively.

Fourier transform infrared spectroscopy (FT-IR) measurements were conducted using FTIR spectrometer (JASCO-6700, Tokyo, Japan), covering the spectral range from 400 to 4000 cm^−1^. An aliquot of 300 μL of Ag-NPs, which was mixed with potassium bromide (10 mg), was oven-dried.

### 2.4. Molecular Identification of MDR Bacterial Isolates

During the period from January 2021 to August 2021, isolates were obtained from urine samples of fifty patients (20 male and 30 female) who were hospitalized at Menoufia University hospitals with UTIs and significant bacteriuria. In accordance with the Declaration of Helsinki, the study received approval from the Ethical Committee of Human Rights of Research at Menoufia University (12/2023MICR4-2). Each participant in this study signed a written informed consent form after being informed about it. All data are stored and maintained. The clinical specimens were streaked on CLED agar plates as soon as they were brought to the Medical Microbiology and Immunology Department’s laboratory. Significantly positive isolates were purified and identified after a 24 h incubation period [36]. Using the Genomic Wizard DNA kit (Promega, Madison, WI, USA), whole bacterial genomic DNA was extracted from all the tested isolates in accordance with the manufacturer’s instructions. The 16S rDNA gene was amplified using two widely used bacterial primers: the RW primer (CCAGCCGCAGGTTCCCCT) and the 16Sb FW primer (CCGTGGCGGCAGGCTTAACA) [37]. The following were the PCR conditions: 35 cycles total—94 °C/30 s for the denaturation step, 55 °C/60 s for the annealing step, 72 °C/90 s for the extension step, and 72 °C/180 s for the final extension step.

The exact dimensions of the amplified PCR product, a fragment of approximately 1500 base pairs, were confirmed by electrophoresis of 10-microliter aliquots on a 1% agarose gel containing 0.5 micrograms per milliliter of ethidium bromide. The gels were inspected and captured on camera using a UV lamp [38]. Next, amplicons were cleaned using a QIAquick spin column (Chatsworth, CA-based Qiagen Inc. (San Diego, CA, USA)). As previously stated, ten purified amplicons were sequenced using a Perkin Elmer 377 DNA sequencer in conjunction with a Dye Deoxy Terminator Cycle Sequencing Kit (Perkin Elmer, Foster City, CA, USA).

### 2.5. DNA Fragmentation Induced by AgNPs MIC

AgNPs were applied at MIC to two isolated bacterial cells to examine DNA fragmentation. The procedure for isolating DNA is shown in the manufacturer’s brochures (Thermo Fisher Scientific, Santa Clara, CA, USA). A DNA purification kit was utilized. After quantification, 4 µg of each DNA sample were electrophoresed on a 1.6% agarose gel that was UV-irradiated and treated with 5 µg/mL ethidium bromide [39].

### 2.6. Minimum Inhibitory Concentration (MIC) and Minimum Bactericidal Concentration (MBC)

The individual strains were cultured in Tryptic Soy Broth (TSB) overnight until they reached an optical density at 595 nm (OD595) of 1. Subculture 1 was 1000 in (TSB). One hundred microliters of bacterial culture were sampled, followed by the addition of 10 microliters of an appropriate serial dilution of AgNPs (NPs/mL) to 96-well plates (F-bottom, Sterilin). The optical density (OD595) was measured using a spectrophotometer called “SPECTRONIC GENESYSTM 2PC”, manufactured by Spectronic, Melville, NY, USA, during a 24 h incubation period at 37 °C.

Forty milliliters of purple p-iodo-nitrotetrazolium (INT, 0.2 mg/mL, Sigma-Aldrich) were added to the microplate wells, and the plates were incubated for an additional 30 min at 37 °C to verify the inhibition of bacterial growth through the lack of metabolic activity [40]. As previously mentioned by Feizi et al. [41], the MIC for the INT assay was found to be the NP concentration that stopped the least amount of color change. Growth was determined by measuring the difference between the OD595 reading and the negative control, Trypticase soy agar (TSA alone), at least two times.

After that, MBC tests were conducted. A 99.9% CFU (3 log) rise in the initial inoculum over the course of a 24 h incubation period was considered bactericidal effectiveness. Fifty ccs from each well of the MIC plate were transferred to a fresh, sterile (TSA) plate, and the MBC was calculated overnight. After a day at 37 °C, viable colonies were counted. This test has a detection limit of 10^1^ CFU/mL.

Using agar well diffusion, the antifungal activity of AgNPs was assessed against *Candida auris* (OR648076) and *Aspergillus niger* (OR648075). Every fungal strain that was evaluated was cultivated on PDA plates and kept at 30 °C for a duration of 3–5 days [42]. After the preparation of the fungal suspension in a sterile phosphate buffer solution (PBS), with pH 7.0, the inoculum was adjusted to 10^7^ spores/mL using a cell-counter chamber. Agar MEA plates were evenly filled with one milliliter. The wells (9 mm) were cut using a sterile cork borer. One hundred µL of AgNPs and AgNO_3_ were then added to each well separately, and the mixture was allowed for two hours at 4 °C. Following the application of a conventional antifungal, chloramphenicol, the plates were incubated at 30 °C for three days. The inhibitory zones were identified and noted following incubation. Additionally, several Ag NP concentrations were assessed for their antifungal properties in order to determine the minimal fungus concentration (MFC).

### 2.7. Antioxidant Activity of the Synthesized AgNPs

A method for measuring antioxidant activity is the DPPH radical-scavenging technique. One method of evaluating the capacity of different plant-leaf extracts to scavenge free radicals is to utilize 1,1-diphenyl-2-picryl hydrazyl (DPPH). In short, an ethanol-based 0.1 mM DPPH solution was prepared. One milliliter of this solution was combined with three milliliters of different extracts in ethanol at different concentrations (3.9, 7.8, 15.62, 31.25, 62.5, 125, 250, 500, and 1000 μg/mL). Here, only extracts that dissolve in ethanol are utilized, and the different strengths of the extracts were made using the dilution process. The mixture was well-shaken and then allowed to stand at room temperature for 30 min. The absorbance was then measured with a UV–VIS Milton Roy spectrophotometer at 517 nm. As the reference-standard compound, ascorbic acid was used in an experiment [43].

% inhibition or DPPH scavenging action = A0 − A1/A0 × 100.

where A1 represented the absorbance while the test or standard sample was present, and A0 represented the absorbance of the control response.

### 2.8. Anti-Inflammatory Effects of AgNPs

#### 2.8.1. Preparation of Erythrocyte Suspension

Three milliliters of freshly drawn blood from healthy participants were centrifuged into heparinized tubes for ten minutes at 3000 rpm. The red-blood-cell pellets were dissolved in a supernatant equivalent to normal saline, quantified, and then reconstituted as a 40% *v*/*v* suspension using an isotonic buffer solution (10 mM sodium phosphate buffer consisting of 0.2 g NaH_2_PO_4_, 1.15 g Na_2_HPO_4_, and 9 g NaCl in 1 L of distilled water). The final pH was adjusted to 7.4 [44].

#### 2.8.2. Hypotonicity-Induced Hemolysis

Distilled water was used to dissolve the extract samples for this test, creating a hypotonic solution. Graded extract dosages (100, 200, 400, 600, 800, and 1000 µg/mL) were added to pairs of double centrifuge tubes (per dose) in the hypotonic solution (5 mL). In addition, pairs of double centrifuge tubes were filled with an isotonic solution (5 mL) containing graded doses of extract (100–1000 µg/mL) (per dose). Five milliliters each of indomethacin 200 µg/mL and distilled water, the excipient, were found in the control tubes. Each tube should contain 0.1 milliliter of red-blood-cell suspension. Gently stir. After 1 h of room-temperature (37 °C) incubation, the mixture was centrifuged for three min at 1300× *g*. Using a spectrophotometer (Milton Roy), the absorbance (OD) of the hemoglobin that makes up the supernatant was measured at 540 nm. The hemolysis ratio was calculated with the assumption that, in the event of distilled water, the resulting hemolysis amount would be 100%. The extract’s hemolysis inhibition ratio was determined [44].

% Hemolysis inhibition = 1 − ((OD2 − OD1)/(OD3 − OD1)) × 100

where OD1 is the test sample’s absorbance in an isotonic solution.

OD2 is the test sample’s absorbance in a hypotonic solution.

OD3 is the control sample’s absorbance in a hypotonic solution.

### 2.9. Phytochemical Analysis

A phytochemical analysis is usually confirmed to evaluate either the presence (positive [+] or absence (negative [−]) of certain chemicals within plant crude extract. Molisch’s test for the presence of carbohydrates is noted by Sofowora [45]. A froth test is used for saponins, according to Mojab et al. [46]. A ferric chloride test is used for tannins, according to Trease and Evans [47]. A biuret test is used for proteins besides amino acids which is described according to Salna et al. [48]. A lead acetate test, a NaOH test, an aluminum chloride test, and a H_2_SO_4_ test for flavonoids according to [23,24]. A ferric chloride test is used for phenols, according to Preshant et al. [49]. A glycosides test and Borntrager’s test is for glycosides, according to Treare and Evans [47] and Harsha et al. [50]. The Keller–Kiliani test and Legal’s test are for cardiac glycosides, according to Balbaa [51] and Preshant et al. [49]. Borntrager’s test (for free anthracene derivatives) and a modified Borntrager’s test (for combined anthracene derivatives) are for anthraquinone, according to Sofowora [45], and Trease and Evans [47] Wagner’s test, Dragendroff’s test, and Hager’s test are for alkaloids, according to [52,53]. An HCl test is for phlobatannins, according to Abdullahi et al. [54]. An alcoholic KOH test and a Ninhydrin test are for quinone, according to Egbuna et al. [55] and Kebede et al. [56]. The test for resins is conducted according to the method described by Egbuna et al. [55].

#### Investigation of Total Active Materials

The total phenolic content (TPC) of the sample was determined using the Folin–Ciocalteu method, as documented by Makkar [57]. Using the aluminum chloride method, the total flavonoid content (TFC) of our plant extract was also determined [58]. The Folin–Ciocalteu method was used to identify and assess the total tannins in the sample [59]. The proportion of saponin content was determined using the methods of Lim et al. [60]. At last, the total alkaloids were assessed using the methodology of Herborne [61], which had been validated.

### 2.10. Static Biofilm Assay

With minor modifications, static biofilms were produced in microtiter plates utilizing the crystal violet staining technique, essentially as previously reported by Saber et al. [62]. Individual strains were grown compactly on TSA plates for a whole night. They were then suspended in TSB and adjusted to an OD595 of 0.02. A 96-well microtiter plate (U-bottom, Sterilin) containing approximately 130 µL of this dilution was incubated for 24 h at 37 °C. Ten μL of AgNP colloidal solution containing 10^5^ NP/mL were added after a 24 h period. As controls, 10 μL of two aqueous plant extracts were added. To evaluate the amount of biofilm, biofilms were dissolved in 96% ethanol and stained with 0.1% crystal violet, and their OD595 was quantified using an Infinite_F50 robotic microplate reader (ostrich).

### 2.11. Ribonucleic Acid Isolation and Complementary Deoxyribonucleic Acid Synthesis

#### 2.11.1. Ribonucleic Acid Isolation

Total ribonucleic acids (RNAs) were extracted from the 72 h fermentation of the *S. aureus* strain, which was cultivated in a liquid nutrient broth medium, through Trizol (Gibcol), according to the manufacturer’s protocol. cDNA for the sense strand was synthesized via the Advantage RT-PCR Kit (Clontech, Alo Alto, CA, USA).

#### 2.11.2. Conventional Reverse Transcription Polymerase Chain Reaction Amplification

Fifty μL reactions of polymerase chain reactions PCR were conducted with primers in Table 1 for the *fnbA* gene within *S. aureus* and Table 2 for the amplification of the *Cna* gene for the same strain. These primers were created using the NCBI site’s Primer BLAST tool. The reference housekeeping gene is *RecA* which also amplified through specific primers listed in Table 3. The PCR reaction mixture consists of 120 ng of synthesized cDNA, 195 mM dNTPs, 0.1 mM of each primer, 1.7 mM Mg Cl_2_, one unit of Taq DNA polymerase (Takara, Tokyo, Japan), and sterile water to adjust the volume to 50 μL. The following describes the PCR program. 

After four minutes of denaturation at 93 °C, there were 29 cycles of 35 s at 93 °C, 45 s at 50 °C, one minute at 45 °C, and a final 6 min extension at 72 °C. A %TAE buffer, which has 1 mM Na-acetic acid (EDTA) and 40 mM Tris-Acetate at pH 7.6, was used to separate the agarose gel. Gels were examined under a UV lamp after electrophoresis and stained with ethidium bromide (0.5 mg/mL). The size was determined using a standard-length DNA ladder (GeneRulerTM 100 bp DNA Ladder, MBI Fermentans, Vilnius, Lithuania).

#### 2.11.3. Real-Time PCR Amplification Conditions

Using the primers listed in Table 1 and Table 2, complementary DNA (cDNA) from the *S. aureus* strain was subjected to semiquantitative PCR. The PCR mixture consisted of 12.5 μL of 2× Quantitech SYBR^®^ Green RT Mix (Fermentase.com), 1 μL of 25 pm/L forward and reverse primers, 1 μL of 50 ng cDNA, and 9.25 μL of RNase-free water. Two primers specific for the *fnbA* gene were found in Table 1, while the two primers specific for *Cna* were found in Table 2. The initial denaturation step in PCR real-time programs lasts for ten minutes at 93 °C for 40 cycles of 15 s. Annealing for 35 s at 45 °C and extending for 30 s at 72 °C.

#### 2.11.4. Data Analysis

Using the dd∆ct technique and Microsoft Excel 2016, a comparative quantification analysis was carried out.

### 2.12. Statistical Analysis

To ascertain the major variations based on many growth characteristics, the data were statistically evaluated. A one-way analysis of variance test utilizing the statistical ANOVA program Spss v17, was used to examine the variables for differences between the various concentrations of the biosynthesized silver nanoparticles caused by variations in the exposure period.

## 3. Results 

### 3.1. Green Synthesis of AgNPs

**Figure 1:**
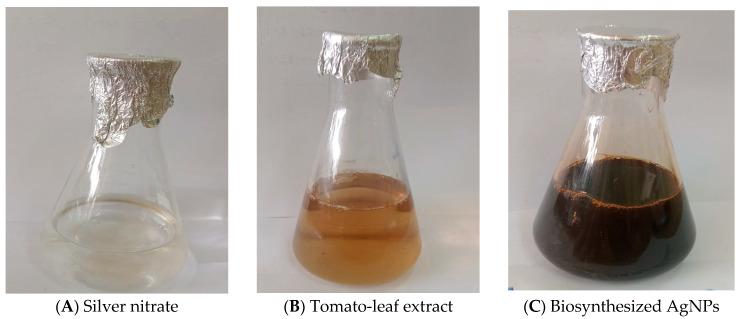
AgNPs were biosynthesized through tomato-leaf extract. Where (**A**) is silver nitrate soln; (**B**) is tomato-leaf extract and (**C**) is the biosynthesized AgNPs.

Tomato-leaf extract was utilized as a reducing agent for the silver nitrate solution during the biosynthesis process. By changing the color of silver nitrate from clear to yellow or deep brown, the creation of NPs was detected (Figure 1). 

### 3.2. Characterization of AgNPs

#### 3.2.1. UV–Vis Spectrophotometry

**Figure 2:**
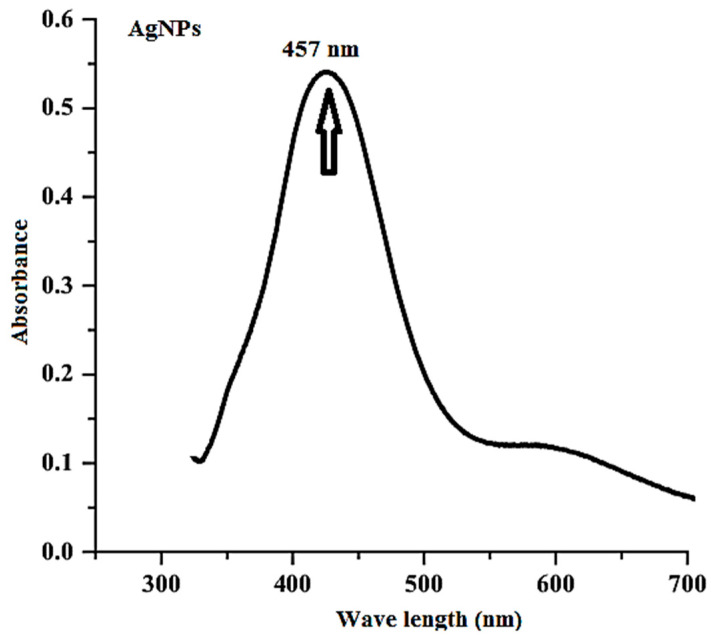
Surface plasmon resonance of biosynthesized AgNPs by using *Solanum lycopersicum*.

AgNPs were characterized by topographical surveys and a physiochemical investigation. Figure 2 shows the UV–visible spectra of AgNPs. The first indication of AgNP production in the UV–Vis spectrophotometer investigation was a color shift to deep brown. Color changes may be attributed to the excitation of the surface plasmon resonance of the produced nanoparticles [64]. With a distinctive peak at 457 nm, surface plasmon resonance SPR provided additional confirmation that Ag had been reduced to AgNPs. The predicted value falls inside the range that was previously mentioned [65,66,67]. 

#### 3.2.2. XRD Analysis

**Figure 3:**
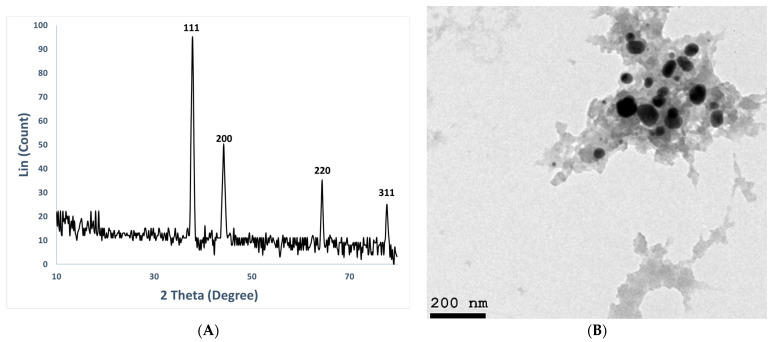
(**A**) XRD analysis and (**B**) TEM imaging of biosynthesized AgNPs.

Biosynthesized NP crystallinity was examined through XRD. Characteristic peaks were determined at 2θ values of 31.7°, 45.6°, 56.3°, and 75.2°, corresponding to the planes (111), (200), (220), and (311). Figure 3A confirms the AgNPs have a face-centered cubic structure. Furthermore, using Debye–Scherrer equation, the crystallite size mean of the synthesized NPs was found to be 55 nm. 

#### 3.2.3. TEM Characterization of Biosynthesized AgNPs

AgNPs were created, and their shape and size were characterized using TEM examination. TEM micrographs (Figure 3B) showed that the particles were almost spherical in shape and monodispersely distributed with little agglomeration. The presence of biomolecules and other metabolites in biomass filtrate, which are utilized in the bioreduction and biocapping of produced nanoparticles, may be the cause of the variation in nanosize and nanoshape [68]. The AgNPs are evenly distributed and do not aggregate, as seen by the TEM picture. The diameters of the nanoparticles varied from 35 to 80 nm, with an average size of 53 nm. 

#### 3.2.4. Fourier Transform Infrared (FT-IR) Spectroscopy

**Figure 4:**
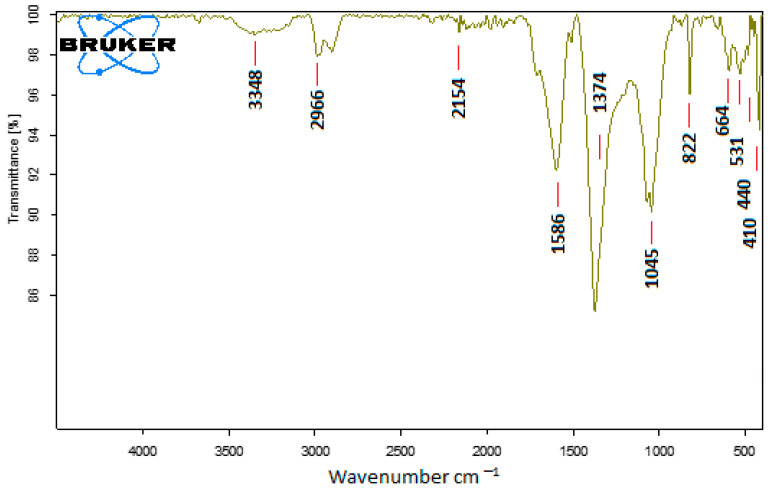
FTIR analysis of biosynthesized AgNPs by using *Solanum lycopersicum*.

To determine the potential biomolecules in charge of the reduction of Ag^+^ ions and capping of the bioreduced silver nanoparticles produced by *Solanum lycopersicum* [*S. cumin*] leaf extracts, FT-IR studies were performed. The FT-IR spectra of the nanoparticles produced are shown in Figure 4. The various functional groups were seen in the FT-IR peaks. Data represented in Figure 4 revealed intense absorption peaks at 3348, 2966, 2154, 1586, 1374, 1045, 822, 644, 531, 440, and 410 cm^−1^. 

### 3.3. Molecular Identification of MDR Isolates

**Figure 5:**
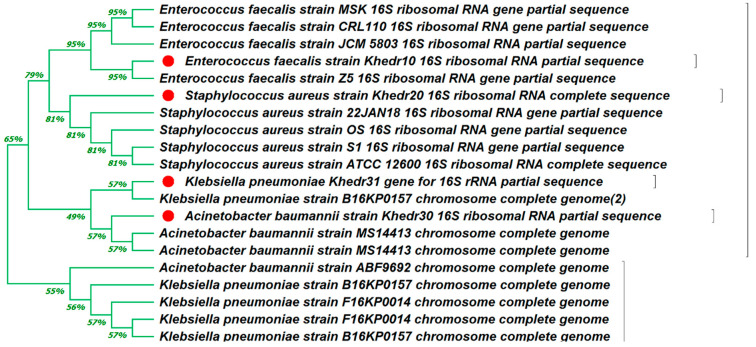
Phylogenetic tree of 16S rDNA-based identification of four MDR bacterial test strains (*Staphylococcus aureus* (OR648079), *Enterococcus faecalis (OR648078)*, *Acinetobacter baumannii* (OR648080), and *Klebsiella pneumonia* (OR648081)) through Mega 11 software (version 11.0.13). The genetically defined organisms are marked with a red circle.

**Figure 6:**
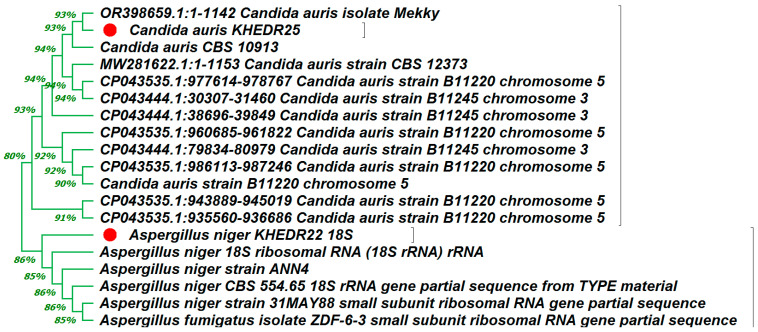
Phylogenetic tree of 18S rDNA-based identification of two MDR fungal test strains (*Aspergillus niger* (OR648075), and *Candida auris* (OR648076)) through Mega 11 software. The genetically defined organisms are marked with a red circle.

Sequencing of the PCR products of the tested MDR bacterial and fungal strains revealed that *Staphylococcus aureus* (OR648079), *Enterococcus faecalis (OR648078)*, *Acinetobacter baumannii* (OR648080), *Klebsiella pneumonia* (OR648081), *Aspergillus niger* (OR648075), and *Candida auris* (OR648076). These phylogenetic trees were built using Mega 11 in comparison to the NCBI BLAST sequences that were the most comparable (Figure 5 and Figure 6). 

### 3.4. DNA Fragmentation

**Figure 7:**
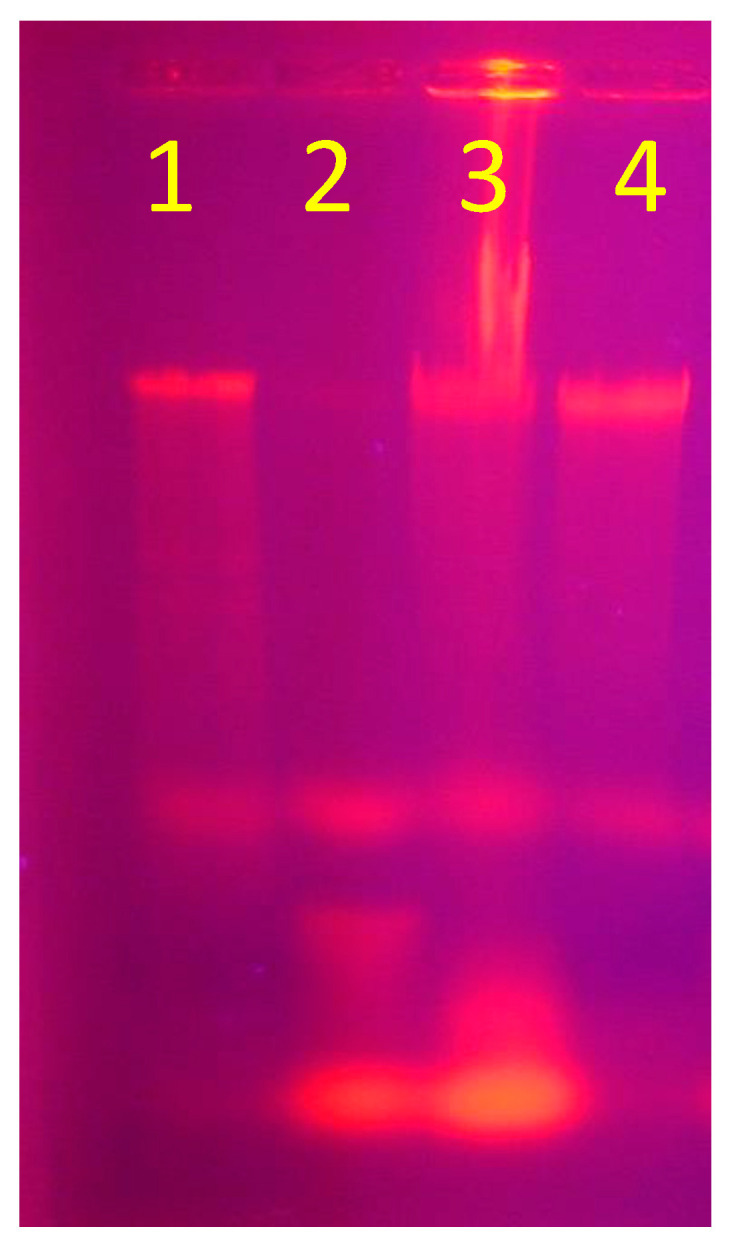
DNA fragmentation of four tested bacterial strains: band 1: *Acinetobacter baumannii*, band 2: *Enterococcus faecalis*, band 3: *Staphylococcus aureus*, and band 4: *Klebsiella pneumonia*.

In our attempt to explain the mechanism by which AgNPs eliminate microbial growth, we used concentrations of AgNPs lower than MIC, MBC, and MFC in order to study their effect on DNA. The findings reported that 3 mg/mL was the best AgNPs concentration, resulting in DNA fragmentation in three bacterial tested strains among four. DNA fragmentation was not noted with *Enterococcus faecalis* alone (Figure 7). Adeyemi et al. [69] noted that DNA damage was induced by AgNPs in addition to oxidative stress. AgNPs cause DNA fragmentation in bacteria, which finally results in cellular death [70].

### 3.5. Antioxidant Assays

**Figure 8:**
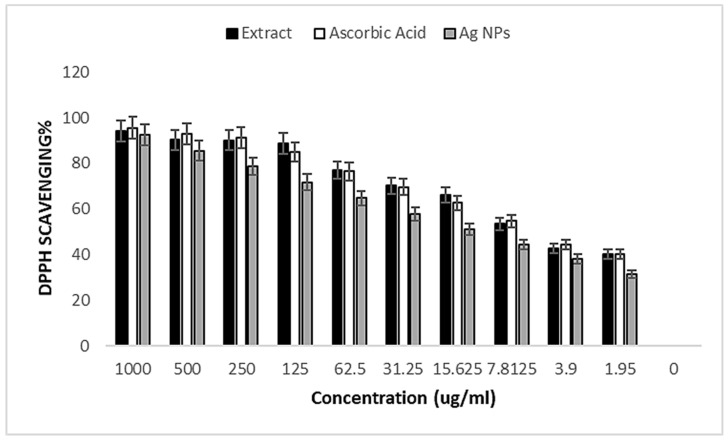
DPPH free-radical scavenging activity of plant extract, AgNPs, and ascorbic acid as references at different concentrations.

Using DPPH and H_2_O_2_ models, the antioxidant capacities of the tomato-leaf extract in an aqueous solution, AgNPs, and standards were investigated. Using ascorbic acid as a reference, the DPPH elimination activity of the studied materials is displayed in Table 1. AgNPs eliminated DPPH significantly in amounts of 92.5, 85.5, 78.7, 71.7, 64.7, 57.8, 51.1, 44.4, 38.1, and 31.4%, which corresponded to an average (IC50 value of 13.54 μg/mL) at various doses (1000, 500, 250, 125, 62.5, 31.25, 15.625, 7.8125, 3.9, and 1.95 μg/mL). These activities, however, paled in comparison to ascorbic acid’s (IC50 3.45 μg/mL). With an IC50 value of 27, the crude plant extract showed a greater inhibitory impact than Ag NP but a lower one than ascorbic acid at the same dosages (Figure 8 and Table 4). 

**Figure 9:**
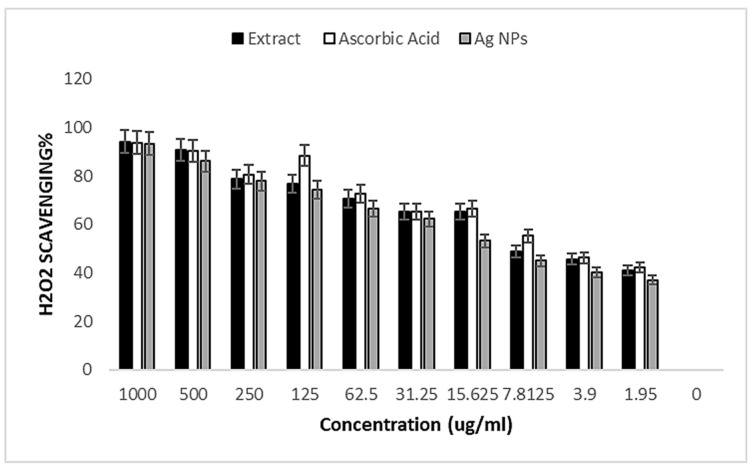
H_2_O_2_ free-radical scavenging activity of plant extract, AgNPs, and ascorbic acid as references at different concentrations.

Using H_2_O_2_ models, the antioxidant capacity of AgNPs, standards, and the aqueous extract of *S. cumin* leaves was investigated. Using ascorbic acid as a reference, the H_2_O_2_ elimination activity of the studied materials is displayed in Table 5. AgNPs eliminated H_2_O_2_ at significant proportions of 93.2, 86.1, 77.8, 74.1, 66.3, 62.3, 53.2, 45, 40.3, and 37.1%, equivalent to an average IC50 value of 13.54 μg/mL at varied concentrations (1000, 500, 250, 125, 62.5, 31.25, 15.625, 7.8125, 3.9, and 1.95 μg/mL). These activities, however, paled in comparison to ascorbic acid’s (IC50 3.45 μg/mL). With an IC50 value of 27.20 μg/mL, the pure plant extract had a greater inhibitory effect than Ag NP and a lower one than ascorbic acid at the same concentrations (Figure 9 and Table 5). 

### 3.6. Anti-Inflammatory Activity

**Figure 10:**
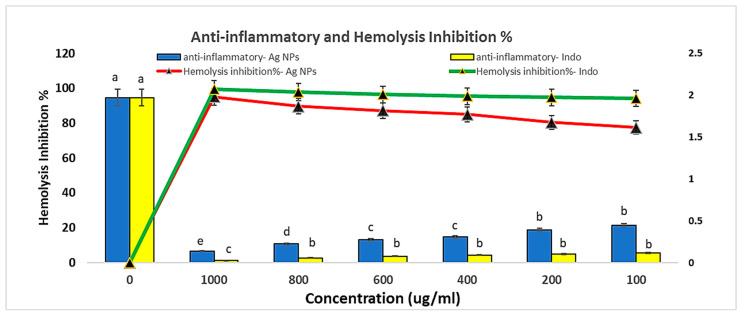
Anti-inflammatory effects of different AgNPs concentrations against reference accompanied by low hemolytic activity. Means with dissimilar superscript letters are significantly different at *p* < 0.05.

The in vitro anti-inflammatory activity was studied using protein denaturation and the (human red blood cell) HRBC membrane hemolysis method. The green synthesized AgNPs were also evaluated for their anticoagulant activity without significant hemolytic activity on RBCs. Different concentrations of AgNPs were tested (1000, 800, 600, 400, 200, and 100 ug/mL), and they recorded 4.8, 10.2, 12.8, 14.9, 19.5, and 22.4 hemolytic inhibition%, respectively. These values were lower than all values recorded with reference to Table 6 and Figure 10. 

### 3.7. Antimicrobial Activity of AgNPs

**Figure 11:**
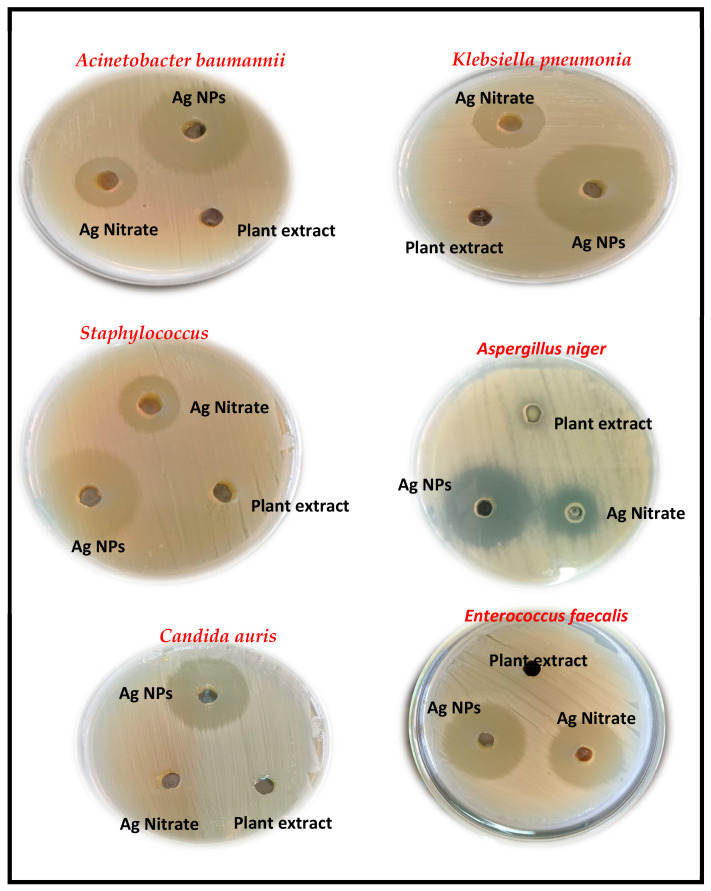
Inhibition zone diameter (mm) in the growth of *Staphylococcus aureus*, *Klebsiella pneumonia*, *Acinetobacter baumannii*, *Enterococcus faecalis, Candida auris,* and *Aspergillus niger* through the agar well-diffusion method for 24 h of incubation against AgNPs, plant extract (*Solanum lycopersicum*), and silver nitrate.

**Figure 12:**
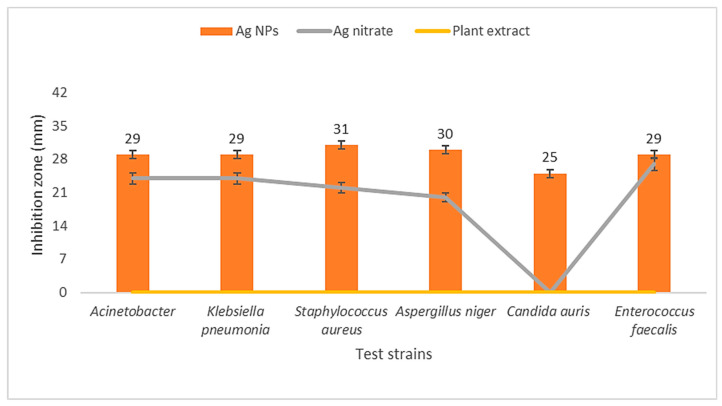
Inhibition zone diameter (mm) of AgNPs, Ag nitrate, and plant extract against five tested MDR bacterial and fungal strains.

**Figure 13:**
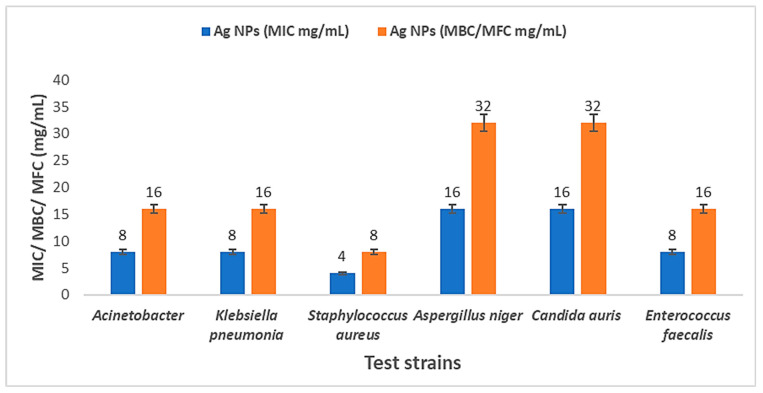
MIC, MBC, and MFC of AgNPs against five tested strains.

AgNPs were effective antibacterial agents against MDR Gram-positive strains such as *Staphylococcus aureus*, Gram-negative strains like *Klebsiella pneumonia*, and *Acinetobacter baumannii,* along with their antifungal effects against *Candida auris* and *Aspergillus niger* (Figure 11). The maximum inhibition activity of AgNPs was demonstrated against *Staphylococcus aureus* with 31 ± 0.81 mm at MIC 4 mg/mL and MBC 8 mg/mL, followed by *Aspergillus niger* with 30 ± 0.53 mm at MIC 16 mg/mL and MFC 32 mg/mL, then *Klebsiella pneumonia*, *Enterococcus faecalis*, and *Acinetobacter baumannii* with 29 ± 0.61 mm, at MIC 8 mg/mL and MBC 16 mg/mL, while the minimal inhibition was against *Candida auris* with 25 ± 0.45 mm at MIC 16 mg/mL and MFC 32 mg/mL (Figure 12 and Figure 13). Ag nitrate has antimicrobial activity, but is lower than AgNPs, with all tested strains except against *Candida auris.* It does not exhibit any inhibitory effect on it. When comparing the inhibitory effects of AgNPs and Ag nitrate through relative percentage, the results showed that AgNPs were more effective than Ag nitrate (20.8%) with *Acinetobacter baumannii* and *Klebsiella pneumonia*. In the case of *Staphylococcus aureus* was 40.9%; in the case of *Enterococcus faecalis*, it was 7.4%. It reaches 50% with *Aspergillus niger*, and it reaches maximum value (100%) with *Candida auris.* Furthermore, AgO NPs at a concentration of 1 μg/mL reduced the growth of several pathogenic bacterial strains, including *Streptococcus* sp., *Bacillus* sp., *Staphylococcus* sp., *E. coli*, *Klebsiella* sp., *Shigella* sp., and *Pseudomonas aeruginosa*, in addition to one fungus, *Candida sp*. The aqueous extract of *Solanum lycopersicum* did not display any significant antimicrobial activity at the tested concentrations. 

### 3.8. Phytochemical Analysis

**Figure 14:**
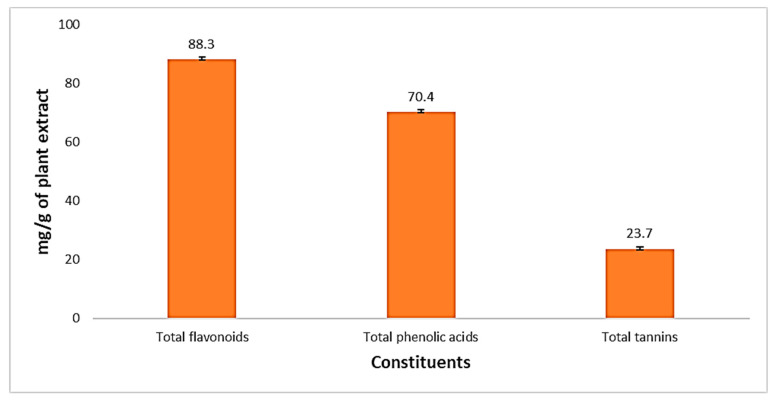
Percentages of total active constituents of *S. cumin* extract through the alcoholic extraction method.

A complete chemical analysis of plant extract was completed after extract preparation, as described by Alsamman et al. [33]. The detection tests for each component, such as carbohydrates, fixed oils, fats, phenol, and tannins, were conducted. It also contains flavonoids and glycosides, in addition to sterols, as summarized in Table 7. Our chemical analysis showed the presence of different active organic components in the plant extract, which concluded in Figure 14 that rutin was 88.3 mg/g, garlic acid was 70.4 mg/g, and tannic acid was 23.7 mg/g. 

### 3.9. Antibiofilm, Cna, and FnbA Virulence Gene Expression

**Figure 15:**
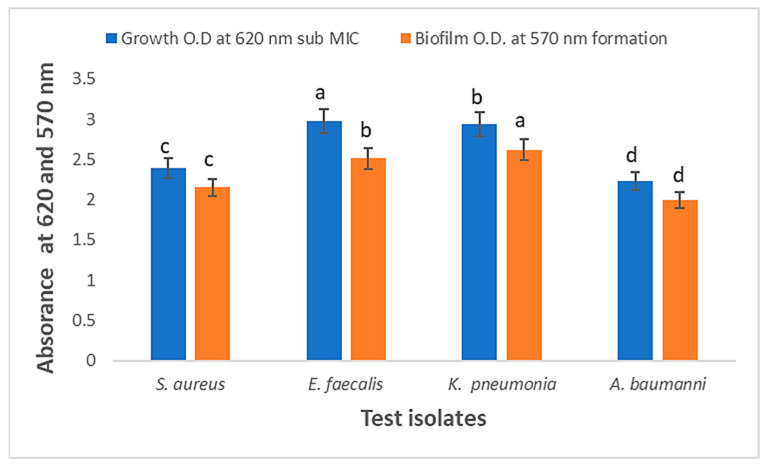
Anti-biofilm formation activity of AgNPs against four MDR isolates through absorbance at 570 nm. Means with dissimilar superscript letters are significantly different at *p* < 0.05.

AgNPs showed high values of anti-biofilm formation activity in comparison with positive control samples against four MDR bacterial isolates. Anti-biofilm was mostly effective against *Acinetobacter baumannii* with 1.99, followed by *Staphylococcus aureus* with 2.15, *Enterococcus faecalis* with 2.51, and finally *Klebsiella pneumonia* with 2.6 (Figure 15).

**Figure 16:**
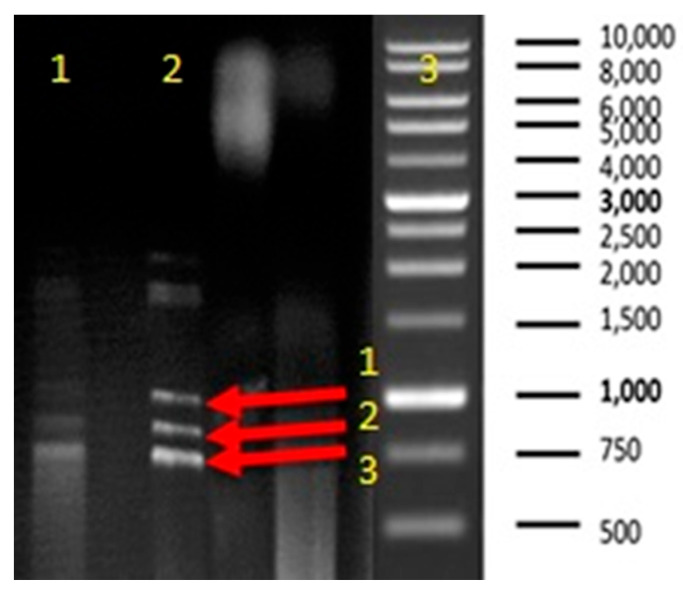
RT-PCR of the three genes within *S. aureus* line [1]: treated sample, line [2]: control sample, wherein both lines of the first band 1 represent *RecA* gene amplicon, the second band 2 represents *Cna* gene amplicon, and, finally, the third band 3 represents *FnbA gene* amplicon.

**Figure 17:**
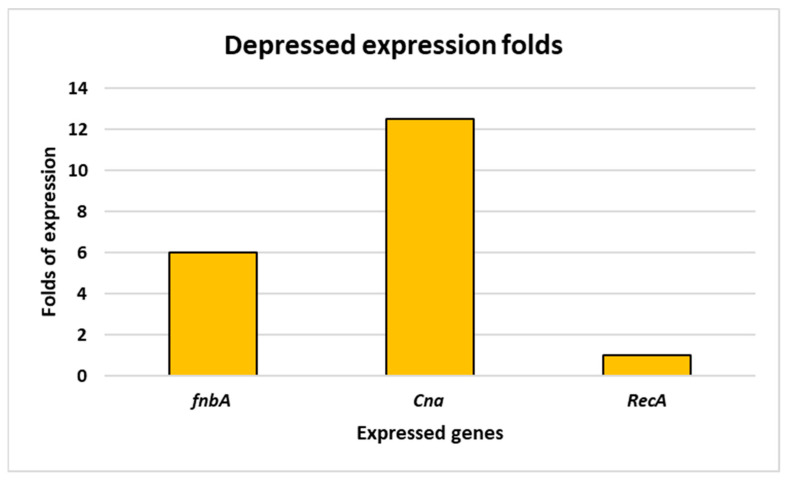
Gene expression in folds for RecA gene against biofilm formation genes *fnbA* and *Can*.

AgNPs had little impact on the biofilm that Gram-negative bacteria formed; their values ranged from 22 to 40.17 mm. Nonetheless, our biosynthesized silver nanoparticles were not as effective against the tested Gram-positive bacteria’s biofilm; 64.85 and 59.13% of the inhibitory effect on biofilm were found against *Candida albicans* and *Candida sake*, respectively; the maximum inhibitory effect was found against Candida species [71]. The high biofilm-suppression activity of silver nanoparticles was demonstrated against *S. aureus*, *Bacillus subtilis*, and *Pseudomonas aeruginosa* [72]. In our research, plant-synthesized AgNPs also cause the downregulation of two fundamental genes for biofilm formation in *S. aureus.* First, total RNA was extracted and then purified, followed by reverse transcription for mRNA. Then, cDNA was used as a template for dsDNA sequences of genes of interest, and a final step aimed at partial sequencing of our genes. A *RecA* partial sequence showed about 1400 bps, *cna* was estimated with 1200 pbs, while *fnbA* was partially estimated at 1K bps (Figure 16). Gene expression exhibits that *fnbA* was depressed in its expression by 6-fold, while another one, *Cna*, was depressed in its expression by 12.5-fold when compared with the housekeeping gene *RecA.* This was analyzed through dd∆ct, in which *RecA* gene expression was used as the standard, as it is one of the housekeeping genes against which were tested two genes of biofilm, *Cna* and *fnbA* (Figure 17).

## 4. Discussion

Plants are thought of as nature’s chemical factories since they are low-maintenance and affordable. Because they generate huge amounts of phytochemicals, a variety of plant components, including fruit, leaves, stems, and roots, have been extensively employed for the environmentally friendly creation of nanoparticles [73]. Environmentally friendly technologies have been used to generate nanoparticles of silver, zinc oxide, and other materials [74,75,76]. Plant extracts contain phytocompounds such as polyols, terpenoids, and polyphenols, which are responsible for metallic ion bioreduction [73]. To lessen the difficulties associated with chemical and physical methods, green techniques (plants, fungi, bacteria, actinomycetes, and yeasts) are preferred for the manufacturing of metal and metal oxide NPs [16,77]. Silver ions were reduced, capped, and stabilized using tomato-leaf extracts. According to Al-Askar et al. [78], combining the zinc acetate solution with the *P. indica* aqueous extract led to the creation of white ZnONPs. Moreover, orange- and pomegranate-peel extracts have greater total phenolic contents, according to Mostafa et al. [79], which supports the reduction of silver ions to AgNPs. Ali et al. [80] biosynthesized nanosilver particles from tomato-peel extract (TPE-AgNPs) and evaluated their characteristics and inhibitory activities against pathogenic bacteria and fungi. According to Singh et al. [81], a leaf extract from *Carissa carandas* L. was used as a reducing and capping agent to accomplish the green biosynthesis of silver nanoparticles (AgNPs). On the other hand, *Rhizopus oryzae* biomass extract was used to biosynthesize silver nanoparticles [64]. Additionally, Iqbal et al. [21] prepared SeNPs by using two types of extracts of freshly prepared tomato (*Solanum lycopersicum*) fruit extract and seed extract for antibacterial and antioxidant activity. Bharathi et al. [82] synthesized FeONPs from *Solanum lycopersicum*-leaf extract and showed potential antibacterial activity against *Escherichia coli*. They also showed significant in vitro anticancer activity against the human lung cancer cell line A549. Elbrolesy et al. [83], investigated the biosynthesis of ZnO NPs using *Solanum lycopersicum*-leaf extract for evaluation of their antibacterial and anticancer activity. Rodríguez-Varillas et al. [84] obtained carbon dots from tomato juice (*Solanum lycopersicum*) in order to obtain nanoparticles with antioxidant capabilities.

The AgNPs’ absorption characteristics are mostly determined by the size and shape of the particles. The typical size of NPs is reflected in the SPR range for AgNPs, which is 380–450 [85]. Nallal et al. [86] proposed the efficient and quick synthesis of AgNPs by sunlight using an extract of *A. ampeloprasum*; the creation of AgNPs was verified by an absorption peak in the UV–Vis spectrum at 446 nm. Singh et al. [81] biosynthesized AgNPs at 432 nm and 444 nm for reactions carried out at 25 °C and 60 °C, respectively. Conversely, higher concentrations of AgNPs and lower average AgNP sizes are linked to higher and lower maximum wavelength values, respectively [87]. El-Bendary et al. [88] detected a peak in the AgNPs spectra at 410 nm, providing evidence that AgNPs were produced by *Aspergillus caespitosus.* Here, Hashem et al. [42] produced AgNPs by B. thuringiensis MAE 6 and had an SPR absorption band at 420 nm. Pallavi et al. [89] reported that the generated AgNPs exhibited a clear and distinct absorption peak at 418 nm, whose broad peak absorption of UV data indicates that the culture supernatant of the *Streptomyces hirsutus* strain SNPGA-8 has reduced silver nitrate to silver nanoparticles.

Furthermore, the XRD analysis is in agreement with Saied et al. [68], who found that AgNPs are biosynthesized using *Cytobacillus firmus* and have diffraction signals (111, 200, 220, and 311). These results agree with those published in [90,91,92], where it was discovered that the average particle size ranged between 5 and 20 nanometers. The average particle size of the AgNPs generated by the *Streptomyces hirsutus* strain SNPGA-8 was 12.74 nm, according to Pallavi et al. [89]. El-Gamal et al. [93] demonstrated that the AgNP formation is shown by strong peaks corresponding to (111), (200), (220), and (311), which were produced by *Streptomyces* sp. Ml-3, and were face-centered cubic in structure and crystallized. According to Ajitha et al. [94], AgNPs synthesized using *Syzygium aromaticum* (clove) extract had the same patterns as AgNPs created under optimal conditions. Four notable diffraction peaks were produced by the AgNPs at 2θ, 38.0°, 44.1°, 64.4°, and 77.4°. These peaks were linked to the lattice planes of the metallic silver’s face-centered cubic (fcc) structure and its crystalline nature.

Additionally, the TEM image is used to detected of shape and size of biosynthesized NPs. The diameters of the nanoparticles of this study were varied from 35 to 80 nm, with an average size of 53 nm. The synthesized BT-AgNPs size varied from 20.15 to 22.21 nm, as demonstrated by Chi et al. [95]. Furthermore, the average size of the AgNPs produced by the extract of walnut fruit (*Juglans regia*) was 31.4 nm [96]. Nagaraja et al. [97] showed that the TEM picture of LnFb-AgNPs was between 10 and 45 nm in size; the TEM micrographs showed that the average particle size was 24.50 nm. Hamouda et al. [98] created spherical AgNPs with diameters ranging from 100 to 200 nm using *Oscillatoria Willlei* NTDM01 extract. On the other hand, the size of spherical AgNPs synthesized by *Bacillus* sp. KFU36 was extended between 5–15 nm [99]. Another study by Lakhan et al. [99] demonstrated, that NPs synthesized using a clove-bud extract had a polydispersed nature and nanoparticle sizes ranging from 10 to 50 nm without agglomeration.

The FTIR analysis was used to detect the functional groups used as a reducing agent for NPs biosynthesis. The broad peak at 3348 cm^−1^ originated from ^−^OH groups in the saponin structure. It is also noted that these bonds may be due to the stretching of ^−^OH in proteins, enzymes, or polysaccharides present in the extract [100]. The peaks at 2966 and 2115 cm^−1^ are due to the C–H stretching of the methylene group or aliphatic group, and it is also a characteristic peak of triterpenoid saponins. The band at 1586 cm^−1^ denoted the bending vibrations of the amide I group and suggested the possibility of binding AgNPs with proteins found in the extracts [81]. Two bands observed at 1374 and 1045 cm^−1^ showed the presence of −C–O stretching of phenol or tertiary alcohols; the band at 1043.27 cm^−1^ showed the O–H stretching of the phenol group [101]. Two bands at 822 and 644 cm^−1^ were because of the C–O stretch and C–S stretch, or the involvement of aliphatic chloro compounds; the band at 644 cm^−1^ might be due to the C–H stretching of the aromatic group; and the peak at 531 cm^−1^ showed the OH group of phenols [102]. Additional information has revealed that proteins may function as stability or capping agents during Ag-NP production [34,103]. According to Singh et al. [81], biomolecules involved in the reduction of Ag^+^ ions and the capping of biosynthesized AgNPs were identified by analyzing the Fourier transform infrared spectra of leaf extract from *Carissa carandas* L., which mediated the production of different silver nanoparticles. An FTIR spectrum study indicates that Meena et al. [100] discovered that the aqueous leaf extract from *Cucumis prophetarum* may act as stabilizing and reducing agents while Ag-NPs are synthesized.

For molecular identification the 16S rDNA technique was used to identify of MDR Isolates. Similarly, Abdelrazik et al. [104] used 16S rDNA PCR sequencing to identify the top five MDR bacterial isolates: *Escherichia coli, Pseudomonas aeruginosa, Acinetobacter baumannii, Enterococcus faecalis*, and *Klebsiella pneumoniae,* with accession numbers OP741103, OP741104, OP741105, OP741106, and OP741107, respectively. Other research conducted by Khedr et al. [105] included the identification of three LAB isolates as *Lactobacillus delbrueckii* using 16S rDNA.

For the antioxidant assay, the interaction of biomolecules with molecular oxygen in biological systems produces free radicals [106]. Consequently, the ROS action is resisted by using antioxidant compounds. The AgNPs effectively showed DPPH inhibition activity, and it was found to be dose-dependent [107]. According to Shehabeldine et al. [108], who reported at doses of 4000, 2000, and 1000 µg/mL, Chi/Ag-NPs had a potential antioxidant activity of 92, 90, and 75%; their IC50 was 261 µg/mL. Several bio-reductive groups of phytochemicals found on the surfaces of Ag-NPs are thought to be responsible for the highest level of antioxidant activity [108]. According to Rajivgandhi et al. [109], the antioxidant values of 0.765, 0.478, and 0.890 were observed at 250 μg/mL. The toxicity effect of AgNPs is determined and influenced by multiple factors; for example, the size-dependent toxicity effect of AgNPs was proven in a study where 10 nm particles portrayed significantly greater toxicity compared to 50 nm, 100 nm, and 200 nm particles. Meanwhile, the shape-dependent toxicity of AgNPs was illustrated in a comparative study on the effect of silver wires (diameter 100–160 nm), spherical silver nanoparticles (30 nm), and silver microparticles (<45 μm) synthesized by wet chemical methods on human alveolar epithelial cells (A549). Based on the results reported, reduction of cell viability and increased lactic acid dehydrogenase (LDH) release by silver wires were observed, while spherical AgNPs showed no such effects. So, we concluded that AgNPs are suitable to be included in a wound-healing formulation up to 25 µg/mL. The total antioxidant activity [110] of the synthetic AgNPs was observed by Dridi et al. [111] to be similar to that of the standard, gallic acid. AgNPs demonstrated a greater capacity to scavenge DPPH than ascorbic acid, indicating their antiradical action (13.08 IP) with an inhibition percentage of 5.09. When compared to ascorbic acid, which had a ferric antioxidant reduction power of 0.66, these manufactured AgNPs show a higher one of 9.46 mg EAA/g DM. Similar findings were reported by Rajeshkumar et al. [112], who discovered that the antioxidant and DPPH free-radical scavenging activities were increased more by the seaweed synthesis of NPs than by crude extract. Pfp-AgNPs biosynthesized by Govindappa et al. [113] had shown better H_2_O_2_ and DPPH scavenging activities, which might have been due to the structure, characterization, and type of capping agents. In another study, the biogenic AgNPs inhibited 69.73 ± 0.56% of DPPH free radicals at 500 μg mL^−1^, indicating considerable antioxidant potential [114].

Another feature of green synthesized AgNPs, aside from the most common ones, is their anti-inflammatory effect [115]. Inflammation is a complicated process that is mostly accompanied by elevated blood vessel permeability, increased protein denaturation, and changes in the cellular membrane. When protein denaturation occurs, they lose their secondary and tertiary structures as a result of heat or stress, resulting in inflammation [116]. Any chemical could be divided into three distinct groups based on its hemolytic impact, according to the American Society for Testing and Materials [117]: hemolytic (hemolysis greater than 5%), mild hemolysis (between 2% and 5%), and no hemolysis (less than 2%) [118]. Using the chemical quercetin, Chahardoli et al. [119] investigated the anti-inflammatory effect of biosynthesized AgNPs and found comparable outcomes. The biosynthesized NPs exhibited 100% anti-inflammatory efficacy, which can be attributed to the coating agent quercetin present on their surface. In another study, protein denaturation is a perfectly documented reason for the inflammation in conditions such as rheumatoid arthritis. The prevention of protein denaturation is the main mechanism of action of nonsteroidal anti-inflammatory drugs (NSAIDs) [120]. The biogenic silver nanoparticles biosynthesized by Sabarathinam et al. [121] show comparable anti-inflammatory activity with standard diclofenac sodium, which is a chemical analgesic at a 50 microL concentration and can act as a potent anti-inflammatory drug. It is commonly known that the mechanism of action of protein denaturation inhibitors is the suppression of several inflammatory mediators involved in the inflammatory process [122]. Drugs including phenylbutazone, salicylic acid, sodium diclofenac, and flufenamic acid exhibit dose-dependent efficacy against protein denaturation, which is a well-documented source of inflammation [123]. Additionally, it has been noted that a variety of plant extracts and the separated chemicals from them had strong anti-inflammatory action that was on par with that of synthetic anti-inflammatory medications. The majority of anti-inflammatory drugs stabilize the plasma membrane of mammalian erythrocytes and thereby inhibit heat-induced and hypotonicity-induced hemolysis [123,124]. Additionally, In vitro, AgNPs are thought to have anti-inflammatory effects, as they play a role in the wound-curing process by TNF-α, interferon, and interleukin-1, as well as by inhibiting COX-2 besides MMP-3 expression. They may diminish the activity of TNF-α, which is involved in inflammatory processes [125,126]. Chi et al. [127] discovered that the kernel-fabricated silver nanoparticles possess a reasonable anti-inflammatory (69.77%).

For antimicrobial activity, The *Enterobacterales* order, which includes Gram-negative bacteria found in the intestine, like *Escherichia coli* and *Klebsiella* spp., is known to cause UTIs. Other pathogens include nonfermenting Gram-negative bacteria like *Pseudomonas* spp. and *Acinetobacter* spp., atypical microorganisms like *Mycoplasma* and *Ureaplasma* species, and yeast (*Candida* spp.) [128,129,130].

Similarly, Loo et al. [131] observed that AgO NPs minimum inhibitory concentration (MIC) against *Salmonella typhimurium*, *Escherichia coli*, *Klebsiella pneumoniae*, and *Salmonella enteritidis* was 7.8, 3.9, 3.9, and 3.9 μg/mL, in that order. AgO NPs’ bactericidal activity has been evaluated using growth curves at doses ranging from 0 to 8×MIC dilution factor [33]. Khaydarov et al. [132] and Yousef et al. [133] reported that the AgNPs MIC values for *S. aurues* and *Enterococcus faecalis* were 3 and 2 mg/mL, respectively. Alsamman et al. [33] reported that AgO NPs exhibit maximum antibacterial activity among the selected five MDR-tested isolates, with TiO_2_ nanoparticles being the second most effective type. Microbial AgO had an average size of 30 nm by TEM with a smooth and regular spherical shape, while plant-mediated AgO NPs had an average size of 52 nm as demonstrated by TEM examination. AgO and TiO_2_ showed notable antibacterial activity for all the MDR isolates tested, especially against *E. coli* and *Staphylococcus sciuri* [33]. The findings, determined by Sharifi-Rad et al. [120], demonstrate the antibacterial activity of the *A. tribuloides* root extract and the greenly synthesized AgNPs against the Gram-positive (*B. cereus* and *S. aureus*) and Gram-negative bacterial strains (*E. coli* and *Sh. flexneri*) at comparable concentrations (500 μg/mL). It was discovered that strains of bacteria were significantly inhibited by the *A. tribuloides* root extract and the environmentally produced AgNPs. In contrast to Gram-positive bacteria strains, AgNPs showed more inhibition action against Gram-negative bacteria, whereas *A. tribuloides* root extract demonstrated greater inhibition activity against Gram-positive bacteria. The TPE-AgNPs from tomato peels (*Solanum lycopersicum*) obtained by Ali et al. [80] showed that *B. subtilis* and *E. coli* were the most sensitive pathogens, with IZDs of 4.0 and 0.92 cm, respectively However, *L. monocytogenes* and *S. sonnei* were the most resistant pathogens, with IZDs of 0.92 and 0.90 cm, respectively. The synthesized TPE-AgNPs from tomato peels had good inhibitory potentials against pathogenic fungi, with IZDs of 3.0 and 0.92 cm against *A. solani* and *C. albicans*, respectively.

The high antibacterial activity of AgNPs is attributable to their large surface area, which provides better contact of the nanoparticles with the cell walls of microorganisms [134]. The biosynthesized AgNPs functioned through the mechanisms of increased affinity for sulfur proteins and electrostatic attraction caused by silver ions that could cling to the cytoplasmic membrane and cell wall. The bacterial envelope may be disturbed due to the associated ions’ potential to enhance the cytoplasmic membrane’s permeability [135]. As lipid peroxidation increases, cellular reducing sugars and total proteins flow through damaged cell membranes [136]. DNA replication and cell reproduction can be affected by the interaction between silver ions and the sulfur and phosphorus in DNA. Silver ions also have the ability to stop the synthesis of proteins by denaturing ribosomes in the cytoplasm [137]. Additionally, the dissolution of AgNPs releases antimicrobial silver ions, which actively interact with thiol-containing proteins within the cellular wall and affect their functionality. Upon interaction with the outer membrane, AgNPs are capable of binding with proteins and forming complexes with electron donors containing oxygen, phosphorus, nitrogen, or sulfur atoms [138,139].

For the phytochemical analysis, a complete chemical analysis of plant extract was examined. The findings reported by Mohammed et al. [140] show that the qualitative phytochemical analysis showed that while carbohydrates, steroids, and anthroquinone were absent from both the healthy and infected leaves of *Solanum lycopersicum*, alkaloids, flavonoids, tannins, cardiac glycosides, phenols, and saponins were present. The quantitative study showed that both healthy and infected leaf curls of *Solanum lycopersicum* contained 8.2% and 3.8% alkaloids, 49.6% and 48.2% flavonoids, 30.6% and 19.99% tannins, 13.6% and 7.022% phenols, and 1.2% and 0.1% saponins. According to Mehmood et al. [141], who evaluated the phytochemicals of black-cumin extracts, they confirmed the occurrence of flavonoids, alkaloids, phenols, and tannins. Also, the bioactive components of the ethanolic extracts of five popular Indian seed spices—coriander, cumin, fenugreek, fennel, and black cumin—were qualitatively analyzed. These extracts’ qualitative phytochemical assays reveal the presence of many phytochemicals, including anthocyanin, steroid, flavonoid, saponin, tannin, alkaloid, and coumarin. The most prevalent phytochemical found in all of the seed spices was an alkaloid. Notably absent are coumarins and steroids, with the exception of black cumin and fenugreek, respectively [142]. According to Balaram et al. [143], who determined the phytochemicals of *Cuminum cyminum*, screening shows the presence of phytochemicals like alkaloids, glycosides, steroids, flavonoids, tannins, saponins, resins, and phenols.

## 5. Conclusions

In conclusion, our study presents a simple and cost-effective method for synthesizing biogenic Ag nanoparticles (NPs) using *Solanum lycopersicum* (*S. cumin*), commonly known as tomato. Through UV–visible spectroscopy, XRD, and TEM analyses, we characterized the biosynthesized AgNPs, observing a maximum surface plasmon resonance (SPR) at 457 nm. TEM imaging revealed particle diameters ranging from 5 to 30 nm, while XRD analysis confirmed their crystalline nature, with an average size of 55 nm. Importantly, these AgNPs exhibited significant antimicrobial activity against a variety of pathogens, including unicellular and multicellular fungi and Gram-positive and Gram-negative bacteria. AgNPs showed inhibition activity against *S. aureus,* with 31 mm at MIC 4 mg/mL and MBC 8 mg/mL, followed by *A. niger* with 30 mm at MIC 16 mg/mL and MFC 32 mg/mL, then *E. faecalis*, *K. pneumonia* and *A. baumannii* with 29 mm, at MIC 8 mg/mL and MBC 16 mg/mL, while the minimal inhibition was against *C. auris* with 25 mm at MIC 16 mg/mL and MFC 32 mg/mL. Additionally, the AgNPs demonstrated antioxidant properties, effectively scavenging DPPH and H_2_O_2_ radicals (AgNPs at different concentrations removed DPPH and H_2_O_2_ at IC50 value of 13.54 μg/mL) and influencing the downregulation of critical genes involved in biofilm formation in *S. aureus*, indicating potent antibiofilm activity. AgNPs concentrations in the range of 3–6 mg/mL showed decreased expression of two of the fundamental genes necessary for biofilm formation within *S. aureus, fnbA* (6-fold) and *Cna* (12.5-fold) when compared with the *RecA* gene, which decreased by one-fold when compared with the control sample. The results showed that both tomato-leaf extract and biosynthesized AgNPs have remarkable antimicrobial, antibiofilm, antioxidant, and anti-inflammatory activity, although the AgNPs effect was greater than that of tomato-leaf extract in these activities. In addition, two genes of biofilm formation in *S. aureus*, *Cna,* and *FnbA* were depressed with AgNPs.

For future research, several avenues are suggested.

1. Further investigation into the mechanisms through which AgNPs exert their antimicrobial and antibiofilm effects could yield valuable insights, enabling the optimization of their efficacy and specificity;
2. Assessing the effectiveness and safety of biosynthesized AgNPs in animal models is essential for the translation of these findings into practical applications;
3. Investigating the synergistic effects of AgNPs with existing antibiotics or antimicrobial agents could present new strategies for combating microbial resistance;
4. Evaluating the potential of AgNPs for environmental disinfection and as coatings for medical devices could broaden their use in preventing microbial contamination and healthcare-associated infections;
5. Further research into how AgNPs affect the expression of genes involved in microbial virulence and resistance mechanisms could improve our understanding of their comprehensive antimicrobial properties.

Overall, the development of biogenic AgNPs from *Solanum lycopersicum* represents a promising path for enhancing antimicrobial strategies, necessitating ongoing research to fully exploit their potential in both clinical and environmental applications.

## Figures and Tables

**Table 1:** Specific primers used to detect the *fnbA* gene in the *S. aureus* strain.

Primer	Sequence (5’->3’)	Tm	GC%	Self 3’ Complementarity	References
**Forward primer-1**	AGCGGTAACCAGTCATTCGAG	60	52	2.0	This study
**Reverse primer-1**	TTGGCGGCGTTGTATCTTCT	60	50	0	This study
**Forward primer-2**	GAAGATACAACGCCGCCAAC	59.9	55	0	This study
**Reverse primer-2**	AGGTTCTTCTTTTGCGGGTG	58.8	50	0	This study

**Table 2:** Specific primers used to detect the *Cna* gene in the *S. aureus* strain.

Primer	Sequence (5’->3’)	Tm	GC%	Self 3’ Complementarity	References
**Forward primer-3**	AAGGTGAACAGGTGGGTCAA	59.08	50	0	This study
**Reverse primer-3**	CACTACTTGTTCCCGCTTCA	57.84	50	1	This study

**Table 3:** Specific primers used to detect the *RecA* gene in the *S. aureus* strain.

Primers	Sequence (5’->3’)	Tm	GC%	Self 3’ Complementarity	References
**FW 1RecA**	GCCCTAATTGGTCCAGGCG	45	44.3	0	[63]
**RW 1 RecA**	ACAACGGCGTTCTCTCCTAT	45	44.4	0	[63]
**FW 2 RecA**	ACACAACGTCATTGCAAATGTGA	45	44.3	1.0	[63]
**RW 2 RecA**	GCCTGGACCAATTAGGGCAT	45	44.3	1.0	[63]

**Table 4:** DPPH free-radical scavenging activity of plant extract, AgNPs, and ascorbic acid as references at different concentrations.

Concentration	Crude Extract	Ascorbic Acid	AgNPs
1000	94.1	95.5	92.5
500	90.2	92.9	85.5
250	90.1	91.1	78.7
125	88.7	84.9	71.7
62.5	77.1	76.4	64.7
31.25	70.2	69.6	57.8
15.625	66.1	62.6	51.1
7.8125	53.5	54.7	44.4
3.9	42.8	44.3	38.1
1.95	40.3	40.2	31.4
0	0	0	0

**Table 5:** H_2_O_2_ free-radical scavenging activity of plant extract, AgNPs, and ascorbic acid as references at different concentrations.

Concentration	Crude Extract	Ascorbic Acid	AgNPs
1000	94.1	93.7	93.2
500	90.5	90.4	86.1
250	78.6	80.6	77.8
125	76.7	88.4	74.1
62.5	70.6	72.5	66.3
31.25	65.1	65.1	62.3
15.625	65.4	66.4	53.2
7.8125	48.8	55.2	45
3.9	45.6	46.2	40.3
1.95	41.1	42.3	37.1
0	0	0	0

**Table 6:** Anti-inflammatory effects of different AgNP concentrations against reference accompanied by low hemolytic activity.

Concentration	Anti-Inflammatory—AgNPs	Anti-Inflammatory—Indo	Hemolysis Inhibition%—AgNPs	Hemolysis Inhibition%—Indo
0	1.972	1.972	0	0
1000	0.135	0.027	95.2	99.6
800	0.225	0.056	89.8	97.8
600	0.272	0.076	87.2	96.5
400	0.307	0.092	85.1	95.5
200	0.393	0.103	80.5	94.8
100	0.446	0.115	77.6	94.2

**Table 7:** The preliminary phytochemical screening of *S. cumin*.

*S. cumin* Extracted by Alcohol		
Constituents	Tests	Results
Carbohydrates	Molisch’s test	+
Fixed Oils and Fats	Saponification test	+
Phenol	Ferric chloride test	+
Tannins	Ferric chloride test	+
Phlobatannins	HCL test	−
Flavonoids	Lead acetate test	+
	AlCL_3_ test	+
Saponins	Froth test	−
Glycosides	Glycosides test	+
	Conc. H_2_SO_4_ test	+
Alkaloids	Dragendroff’s test	−
	Wagner’s test	−
	Hager’s test	−
Sterols	Salkawskis test	+
Cardiac glycosides	Legal’s test	−
	Keller Killini test	−

(+ve) mean present, (−ve) mean absent.

## Data Availability

The data presented in this study are available on request from the corresponding author.
